# Interleukin‐12: Central Roles in Immune Regulation and Disease Therapy

**DOI:** 10.1111/imcb.70167

**Published:** 2026-09-30

**Authors:** Anthi Psoma, Geert van den Bogaart

**Affiliations:** ^1^ Department of Molecular Immunology, Groningen Biomolecular Sciences and Biotechnology Institute University of Groningen Groningen the Netherlands

**Keywords:** autoimmune diseases, bacterial infections, IL‐12 immunotherapy, interleukin‐12

## Abstract

Interleukin‐12 (IL‐12) is a key immunoregulatory cytokine produced primarily by antigen‐presenting cells. During infection, IL‐12 orchestrates innate immune responses and shapes the nature of subsequent adaptive immunity. Specifically, by promoting interferon‐γ (IFN‐γ) production in helper and cytolytic T cells and natural killer (NK) cells, it promotes the activation of these cells and drives the differentiation of CD4^+^ T cells into type 1 T helper (Th1) cells. IL‐12 is a heterodimeric cytokine composed of the p35 and p40 subunits, whose coordinated expression is required for formation of the bioactive p70 molecule. The promiscuous use of these subunits within the IL‐12 cytokine family, where p40 and p35 also pair with other partners to form IL‐23, IL‐27, and IL‐35, creates structural and functional overlap that enables fine‐tuned immune regulation. Given its potent immune functions, it is not surprising that IL‐12 availability is regulated at multiple levels, including gene transcription, subunit assembly, and shared receptor signaling components. In this review, we provide an overview of IL‐12, how it is produced and secreted and its mechanisms of action. We also discuss the targeting of IL‐12 for the treatment of various diseases, including bacterial infections, autoimmune diseases, and cancer.

## Graphical abstract

IL‐12 levels are tightly regulated to balance effective protective immunity against infections and cancer with the risk of excessive inflammation and autoimmune disease. Both insufficient and excessive IL‐12 activity can therefore be detrimental, highlighting the importance of precisely controlling its production and signaling.

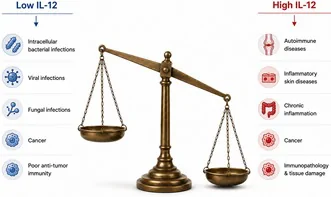

## 1 IL‐12 Biology

Interleukin‐12 (IL‐12), also termed IL‐12p70, is a heterodimeric cytokine with a pivotal role in immune regulation. It consists of two subunits that are linked covalently by disulfide bonds: the α‐chain (IL‐12p35; p35) and the β‐chain (IL‐12p40; p40), encoded by the *IL12A* and *IL12B* genes, respectively [1]. IL‐12 exerts its biological effects through a high‐affinity receptor complex composed of the IL‐12Rβ1 (gene *IL12RB1*) and IL‐12Rβ2 (gene *IL12RB2*) subunits [2, 3]. The p40 subunit associates with IL‐12Rβ1, while the p35 subunit engages IL‐12Rβ2, together forming the functional signaling complex [4]. The p40 subunit of IL‐12 is also capable of forming a homodimer linked by disulfide bonds, referred to as p40₂ or IL‐12p80. p40₂ acts as a functional antagonist of IL‐12 and IL‐23 by binding to IL‐12Rβ1 without triggering downstream signaling [4].

In addition to IL‐12 and p40₂, the IL‐12 cytokine family includes the cytokines IL‐23, IL‐27, and IL‐35 [5, 6]. These are all heterodimers formed by the pairing of an α‐subunit and a β‐subunit that are structurally related to p35 or p40 (Figure 1) [11]. IL‐23 is composed of p19 (IL‐23α) with p40; IL‐27 comprises p28 (IL‐27α) and the Epstein–Barr virus‐induced gene 3 (EBI3) β‐subunit; and IL‐35 is formed by the combination of p35 with EBI3 [12]. IL‐23 signals through a heterodimeric receptor complex consisting of IL12Rβ1 and the IL‐23 receptor (IL23R) [13]. For IL‐35, the p35 subunit binds to IL‐12Rβ2, whereas EBI3 interacts with the gp130 receptor chain [14]. The EBI3 subunit of IL‐27 also binds to gp130 but now in complex to IL27Rα which interacts with p28 (Figure 2) [15].

**FIGURE 1:**
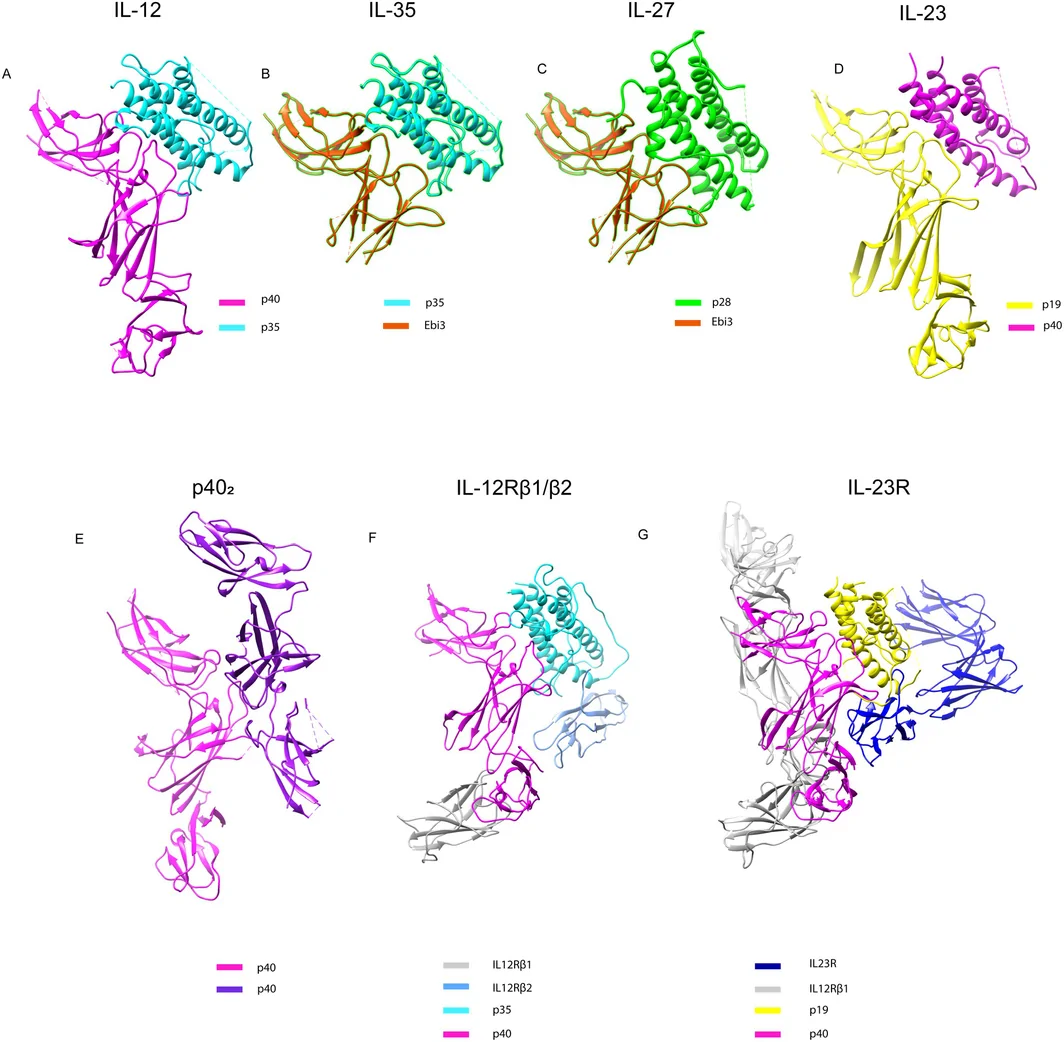
Structural homology of the IL‐12 family and their receptors: (A) Structure of the IL‐12 cytokine consisting of p35 and p40 subunits (PDB: 8CR6) using X‐RAY with 2.85 Å resolution [7]. (B) Structure of the IL‐35 cytokine consisting of p35 and Ebi3 subunits, modeled from PDB: 8CR6 and PDB: 8XWY with Chimera. (C) Structure of the IL‐27 cytokine consisting of p28 and Ebi3 subunits (PDB: 8XWY) using X‐RAY with 3.40 Å resolution (to be published). (D) Structure of the IL‐23 cytokine consisting of p19 and p40 subunits (PDB: 5MXA) using X‐RAY with 2.50 Å resolution [8]. (E) Structure of p40_2_ (PDB: 6SFF) using X‐RAY with 2.40 Å resolution [9]. (F) The Cryo‐EM structure of the IL‐12 receptor subunit beta‐1 and receptor subunit beta‐2 complex (PDB: 8YI7) with a resolution of 3.57 Å binding to the IL‐12 cytokine [10]. (G) The Cryo‐EM structure of IL‐23, receptor subunit beta‐1 and the IL‐23R complex (PDB: 8OE4) with a resolution of 3.60 Å binding to the IL‐23 cytokine composed of p40 and p19 subunits (PDB: 5MXA) modeled by Chimera [7].

**FIGURE 2:**
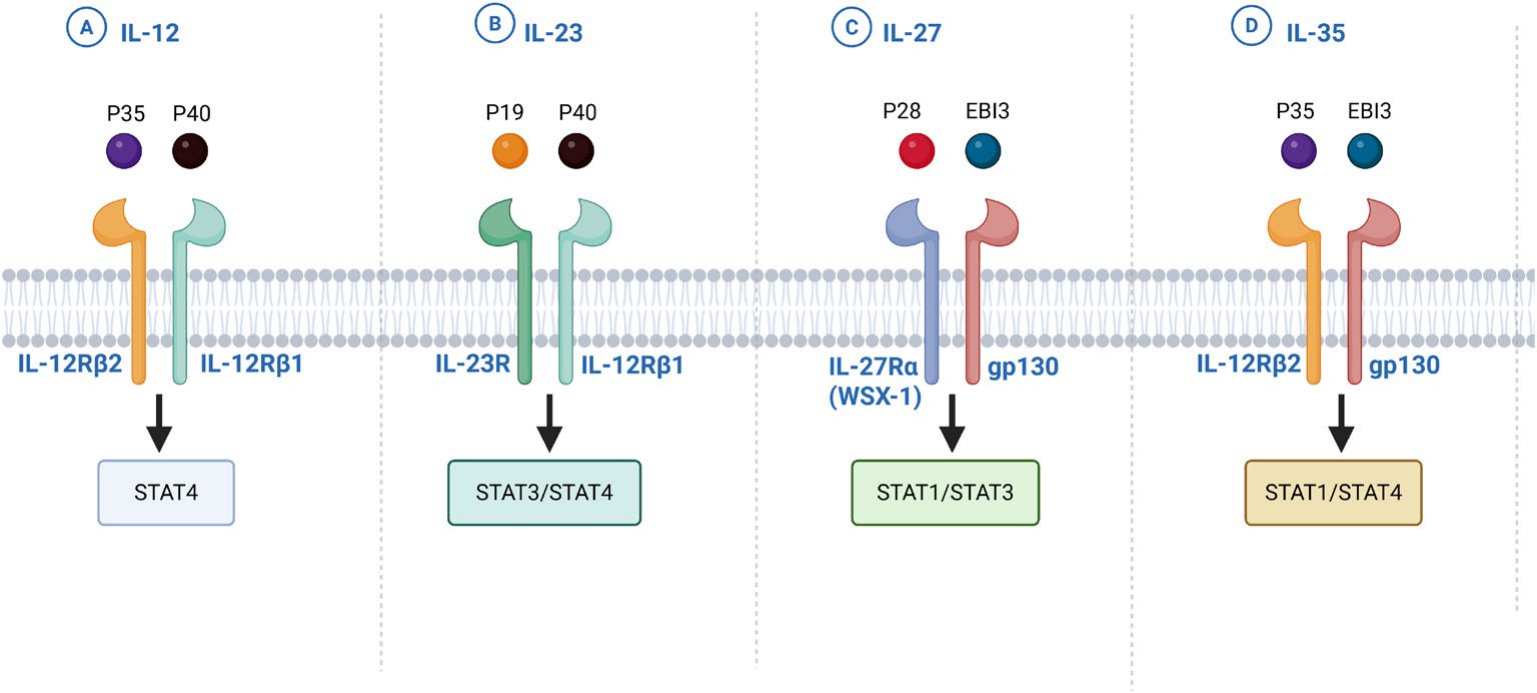
IL‐12 cytokine family members and their corresponding receptor complexes. The IL‐12 cytokine family comprises four heterodimeric cytokines that share structural subunits and receptor chains. The members of the IL‐12 cytokine family are shown together with their corresponding receptors and downstream JAK–STAT signaling pathways.

IL‐12 is produced primarily by activated monocytes, macrophages, neutrophils, B cells, and dendritic cells (DCs) [16]. Regulatory T cells (Tregs) produce the p35 subunit to form the anti‐inflammatory IL‐35 cytokine [17]. Some non‐immune cells, for example, keratinocytes, vascular smooth muscle cells, and fibroblasts, can also express p35 and p40 [18, 19, 20].

During infection, IL‐12 coordinates innate immune defenses and directs the development of the subsequent adaptive immune response. It stimulates interferon‐γ (IFN‐γ) production and promotes the differentiation of CD4^+^ T cells into type 1 T helper (Th1) cells and activation of cytotoxic T lymphocytes (CTLs) and NK cells [21, 22]. Mice lacking p40 or either chain of the IL‐12 receptor (IL‐12Rβ1, IL‐12Rβ2) display markedly impaired Th1 responses and defective pathogen clearance [23, 24]. In studies with *Mycobacterium tuberculosis* , p40 knockout mice exhibited higher bacterial loads and increased mortality compared with p35 knockout mice, indicating that the absence of IL‐23, which requires p40, is particularly detrimental. These mice also display impaired antigen‐specific IFN‐γ production and defective CD8^+^ T cell responses [25]. The loss of IL‐12 signaling blocks Th1 cell development, causing markedly reduced IFN‐γ production, while the absence of IL‐23 signaling impairs Th17 responses, which are critical for defense against certain bacterial infections [26, 27].

In humans, mutations in the *IL12B* and the *IL12RB* genes correlate with reduced ability to eliminate intracellular bacteria, including *M. tuberculosis* and environmental mycobacteria, a condition known as Mendelian susceptibility to mycobacterial disease (MSMD). Clinically, this can manifest as a spectrum of symptoms ranging from severe, disseminated, and often fatal infections in early childhood, typically associated with complete receptor deficiency, to more localized, recurrent infections in children [28].

## 2 IL‐12 Genes and Structure

The human *IL12A* and *IL12B* genes are located on chromosomes 3 (*IL12A*) and 5 (*IL12B*) [29]. The canonical p35 isoform contains 197 amino acids and has a molecular weight of 22.5 kDa without its 22‐residue signal peptide and without glycosylation. The canonical p40 isoform consists of 306 amino acids and has a molecular weight of 34.6 kDa without its 22 amino acid signal peptide and without glycosylation. The genes encoding the IL12 subunits in mice, *Il12a* and *Il12b*, are also located on different chromosomes: chromosome 11 (*Il12b*) and chromosome 3 (*IL12a*) [30]. Murine p35 is 193 amino acids and p40 is 313 amino acids long without glycosylation and signal peptides (Figure 3).

**FIGURE 3:**
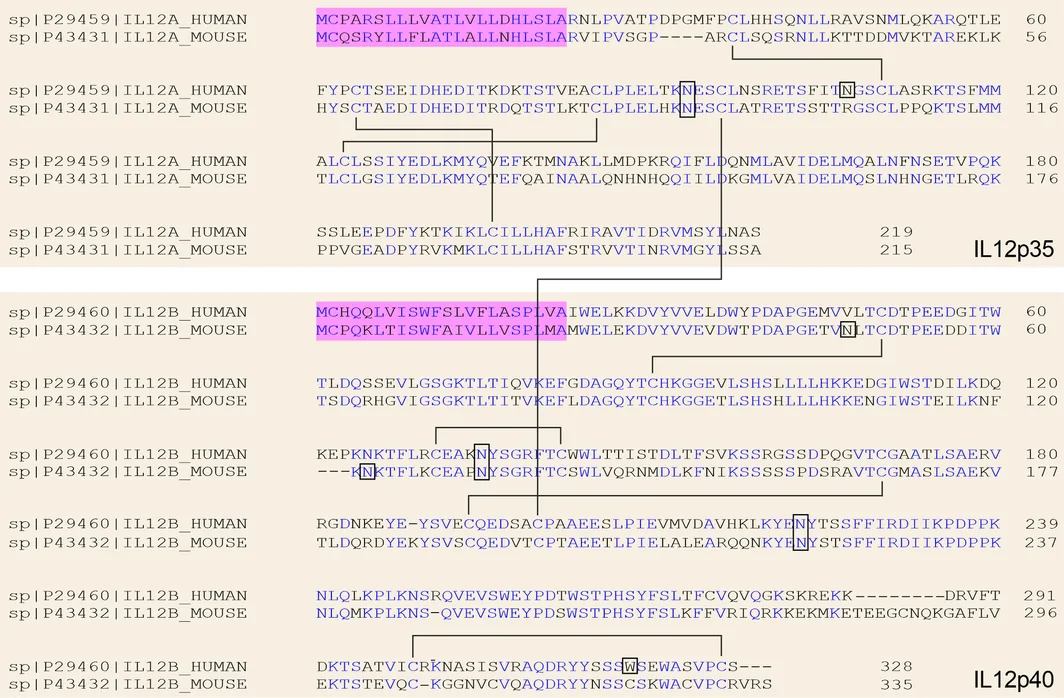
Comparison of conservation of IL‐12. Box 1: Human with mouse p35; Box 2: Human with mouse p40. Boxes: Glycosylation sites, lines: Disulfide bonds, magenta region: Signal‐peptide sequence.

Human *IL12A* has three potential splicing isoforms mapped to its entry, while *IL12B* has two potential isoforms mapped. However, only two splicing isoforms of *IL12A* have been reported experimentally, characterized by the presence or deletion of exon 3. This exon 3‐deficient transcript was detected in both DCs and Epstein–Barr virus‐transformed B cells, and its expression was observed only under conditions of high p35 transcription [31]. Mouse *Il12a* has only a single splicing isoform mapped to its entry, while *Il12b* has at least two isoforms through alternative splicing in exon 5 [32]. In addition, multiple transcription start sites were identified in the murine B‐cell lymphoma line A20 and in bone marrow‐derived DCs, producing four p35 mRNA isoforms (types I–IV) that differ in the number and position of upstream ATGs in their 5′ untranslated regions. In unstimulated cells, the predominant isoforms (types II and IV) contained an upstream ATG that inhibited translation of the p35 subunit. Following LPS stimulation, transcription shifted to alternate start sites, increasing the proportion of transcripts lacking this inhibitory ATG (types I and III), which efficiently supported p35 translation and incorporation into bioactive IL‐12 [33]. Whether a similar regulatory mechanism governs p35 gene expression in humans remains unknown.

The p35 subunit adopts a four‐helix bundle conformation, a structural feature common to class I cytokines. The p40 subunit is composed of three distinct domains: an N‐terminal immunoglobulin‐like (Ig) domain (D1) and two fibronectin type III (FN‐III) domains (D2 and D3). Subunit p35 cannot fold correctly on its own and newly synthesized p35 without p40 or EBI3 forms improper disulfide bonds that lead to misfolding and subsequent degradation. Coexpression of p40 or EBI3 stabilizes p35, promotes proper disulfide bond formation, and facilitates assembly and secretion of the biologically active IL‐12 or IL‐35 heterodimer, respectively. Specific cysteines of p35 are required for proper folding and heterodimerization with p40, whereas others contribute to aberrant disulfide bonding, leading to misfolding and targeting of the protein for degradation [34].

The expression of the *IL12A* and *IL12B* genes is differentially regulated in different cell types. For *IL12A*, IRF1 is required for IFN‐γ–dependent activation of the exon 2 promoter [35], and a κB site within the exon 1 promoter is necessary for c‐Rel–dependent (part of the NF‐κB signaling cascade) induction of IL‐12 by microbial stimuli [36]. Macrophages derived from *Irf1* knockout mice exhibit a selective defect in *Il12a* mRNA synthesis, while transcription of *Il12b* remains intact. This central role in transcriptional activation of *Il12a*, but not *Il12b*, by IRF1 is caused by its direct binding to an inverted IRF response element within the *Il12a* promoter following IFN‐γ stimulation. In addition, IRF1‐dependent activation of *Il12a*'s exon 2 promoter requires cooperation with two adjacent Specificity Protein 1 (Sp1) binding sites [37]. Epigenetic remodeling also plays an important role in *IL12A* expression: In the human THP‐1 monocytic cell line, a nucleosome positioned over the Sp1 binding sites undergoes remodeling following stimulation with LPS and IFN‐γ [38].

Similar to *IL12A*, the promoter region of the *IL12B* gene contains multiple transcription factor binding sites, including those for NF‐κB and Erythroblast Transformation‐Specific (Ets) family members. siRNA silencing of the NF‐κB p50 subunit in human monocyte‐derived DCs showed reduced *IL12B* transcription and IL‐12 secretion without affecting phenotypic maturation or expression of HLA‐DR and costimulatory molecules [39]. As the transcription factors regulating *IL12B* partly differ from those regulating *IL12A*, the selective production of individual IL‐12 family cytokines is determined by the engagement of specific TLRs. For example, activation of TLR4 in human DCs promotes the production of both IL‐12 and IL‐23, whereas activation of TLR2 induces IL‐23 but not IL‐12 [40]. A recent paper showed that combinations of two pattern‐recognition receptor ligands can synergistically enhance the production of IL‐12; therefore, specific combinations of multiple vaccine adjuvants could be more effective than individual immune stimuli alone [41].

## 3 IL12R Genes and Structure

*IL12RB1* is encoded on chromosome 19 in humans and codes for a 639 amino acid protein of 70 kDa without the signal peptide and glycosylation. It is a transmembrane protein with a 516 amino acid extracellular domain responsible for the interaction with p40 [2, 42]. *IL12RB2* is located on chromosome 1 in humans and is translated into an 839 amino acid protein of 94.4 kDa without the signal peptide and glycosylation. Its 595 amino acid extracellular domain interacts with p35 [2, 43]. In mice, the *Il12rb1* gene is located on chromosome 8, whereas *Il12rb2* is located on chromosome 4.

Biochemical and structural studies have established a modular mode of receptor recognition in which IL‐12Rβ1 engages the shared p40 subunit, whereas IL‐12Rβ2 predominantly interacts with the IL‐12‐specific p35 subunit [2, 3, 7, 10]. IL‐12Rβ1 recognizes p40 through its N‐terminal fibronectin type III domains, while the N‐terminal Ig‐like domain of IL‐12Rβ2 engages p35. Recent structures of the complete IL‐12 receptor complex have confirmed this organization and revealed conformational flexibility within the cytokine‐receptor complex assembly [7, 10]. This modular architecture explains how the IL‐12 cytokine family can share molecular components, while maintaining signaling specificity. The common p40 subunit enables both IL‐12 and IL‐23 to recruit IL‐12Rβ1, whereas their distinct α‐subunits engage IL‐12Rβ2 or IL‐23R, respectively. Thus, this structural organization explains how the IL‐12 family can reuse cytokine and receptor components, while still generating distinct biological signals.

IL‐12 binding to its receptor triggers phosphorylation of members of the Janus kinase (JAK) family of protein tyrosine kinases in human T lymphocytes. Specifically, it promotes the tyrosine phosphorylation of tyrosine kinase 2 (TYK2) and JAK2 [44]. Activation of the kinases leads to phosphorylation at tyrosine 693 of Signal Transducer and Activator of Transcription 4 (STAT4), which promotes its dimerization, nuclear translocation, and subsequent regulation of gene expression in T cells [45, 46]. Activation of NF‐kB and STAT4 are critical regulators of Th1 and CTL differentiation [47].

IL‐12 receptor expression has been demonstrated in several immune cell populations. In human T cells, IL‐12Rβ2 is selectively expressed by Th1 cells and is induced during Th1 differentiation, thereby conferring responsiveness to IL‐12 [48]. Functional IL‐12 receptor expression has also been demonstrated in human monocyte‐derived DCs, which express both IL‐12Rβ1 and IL‐12Rβ2 and activate downstream signaling in response to IL‐12 [49]. Similarly, human B cells express functional IL‐12 receptors and respond to IL‐12 [50]. IL‐12 receptor expression has also been reported in human microglial cells, where IL‐12 stimulation induces functional responses and promotes IL‐12 production [51]. In addition, expression of IL‐12 receptor components has been found in NK cells and macrophages, as shown by studies examining IL‐12Rβ2 expression and IL‐12 responsiveness in these populations [52].

The expression of IL‐12 receptors is regulated differently between cell types. For example, whereas naive T cells do not express IL‐12Rβ2, resting NK cells express both IL‐12Rβ1 and IL‐12Rβ2 subunits, allowing them to respond directly to IL‐12 stimulation. IL‐2 further enhances this responsiveness by upregulating IL‐12Rβ1 and IL‐12Rβ2 expression and by increasing STAT4 levels. NK cells from cancer patients receiving low‐dose IL‐2 therapy show elevated expression of IL‐12 receptor chains, suggesting that IL‐2 potentiates IL‐12 responsiveness [53]. Expression of IL‐12Rβ2 in CD4^+^ T cells is tightly regulated by activation signals and cytokines, with notable differences between mice and humans. In both species, IL‐12Rβ2 is absent on naive T cells and is induced upon T cell receptor activation [54]. However, in mice, *Il12rb2* expression is primarily driven by IFN‐γ, whereas in humans, expression of *IL12RB2* is driven by IFN‐α, although IFN‐γ can also enhance its expression [55].

## 4 Evolutionary Conservation of IL‐12 and Its Receptors

IL‐12's key role in the immune system is apparent from the high level of evolutionary conservation of IL‐12 and its receptors (Figure 1). Phylogenetic analyses indicate that both IL‐12‐family cytokines and their receptors have deep evolutionary origins. Core ligand subunits, particularly p35, p40, and EBI3, are broadly conserved across vertebrates, and multiple p35 and p40 paralogues have been identified in teleost fish, reflecting expansion through genome‐duplication events [56, 57]. IL‐12‐family receptor components are similarly ancient; large‐scale comparative analyses suggest that ancestral IL‐12 receptor‐like proteins arose early in metazoan evolution [58]. Functional IL‐12Rβ1 and IL‐12Rβ2 orthologues have also been characterized in teleosts, supporting conservation of IL‐12 receptor signaling across vertebrates [59]. Overall, the conservation of core ligand and receptor components suggests that IL‐12‐family signaling represents an ancient immune regulatory system that was subsequently diversified to support increasingly specialized immune functions.

Human and mouse IL‐12 share 60% sequence identity in the p35 subunit and for p40 there is a 70% identity [60]. Nevertheless, despite this evolutionary similarity, IL‐12 displays one‐way species specificity: Mouse IL‐12 is biologically active on human cells, whereas human IL‐12 shows little to no activity on mouse cells [61]. For studying the efficacy and toxicity of human IL‐12 in vivo, humanized mouse models with knock‐in of the human *IL12RB1* and *IL12RB2* genes have been developed [62].

## 5 Secretion of IL‐12

Beyond serving as a structural component of the IL‐12 heterodimer, p40 also contributes to the post‐translational maturation of p35. When expressed alone, p35 is relatively unstable and prone to misfolding in the ER through the formation of non‐native disulfide bonds, resulting in intracellular retention and degradation [34, 63]. Association with p40 promotes assembly‐induced folding of p35, stabilizes the subunit, and facilitates its trafficking through the Golgi and subsequent secretion as bioactive IL‐12p70 [34, 63]. In this context, p40 has been proposed to exert a chaperone‐like function by promoting the proper folding and maturation of p35, although this activity differs from that of classical molecular chaperones [64]. Together with the distinct signal‐peptide processing and glycosylation requirements of p35, this assembly‐dependent mechanism represents an important post‐translational quality‐control checkpoint in IL‐12 biogenesis and secretion (Figure 4) [36, 65].

**FIGURE 4:**
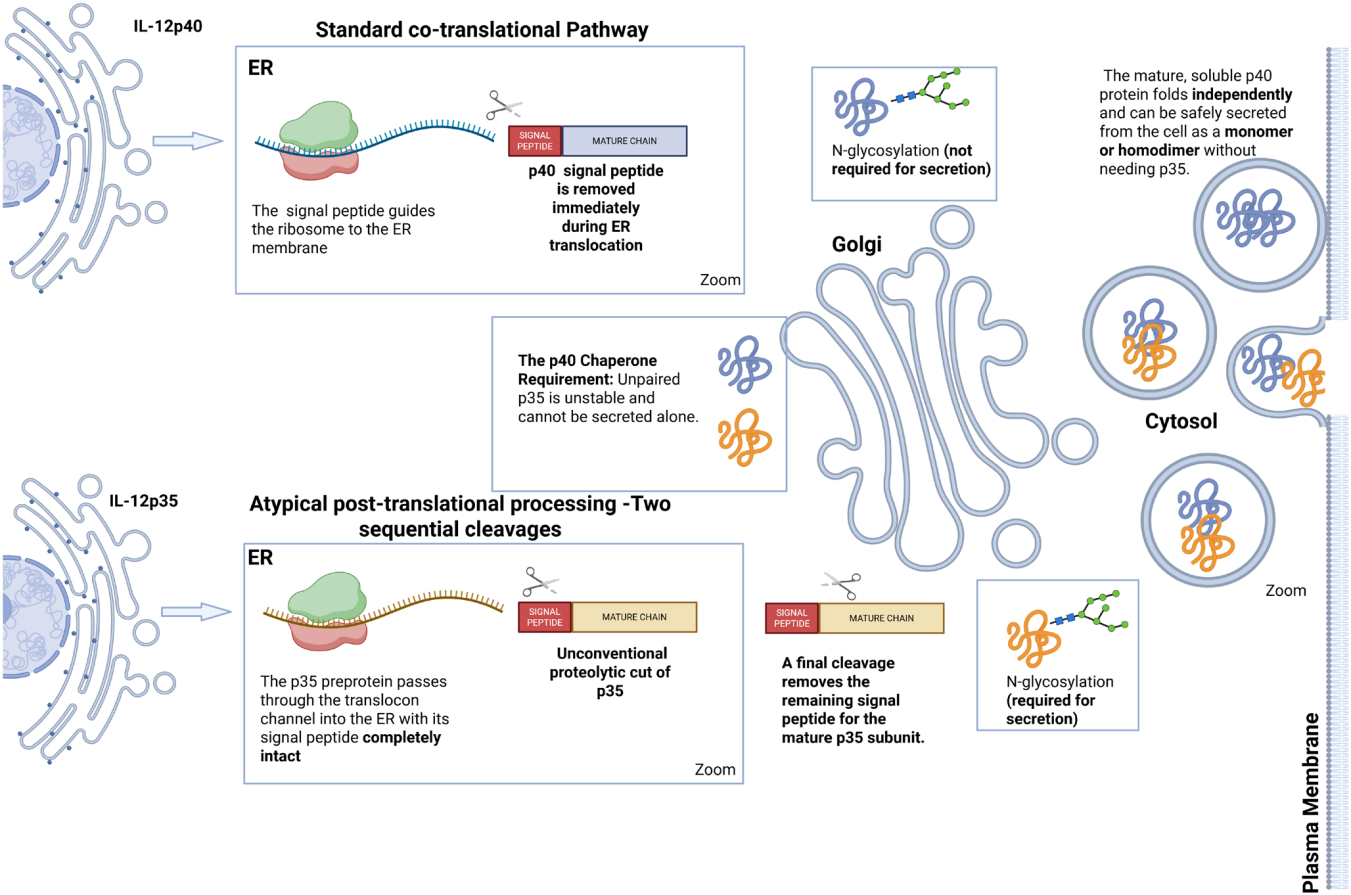
Intracellular processing, assembly, and secretion of the bioactive IL‐12p70. The IL‐12 subunits p40 (blue) and p35 (orange) enter the secretory pathway through the endoplasmic reticulum (ER), where they undergo distinct signal‐peptide processing and post‐translational maturation. p40 follows the standard co‐translational pathway, with cleavage of its signal peptide during translocation at the ER. In contrast, p35 undergoes atypical post‐translational signal‐peptide processing involving sequential cleavage events and requires glycosylation for efficient secretion. Within the ER, association of p35 with p40 acting as a chaperone, promotes proper folding and stabilization of p35, enabling formation of the bioactive IL‐12p70 heterodimer. The assembled cytokine subsequently traffics through the Golgi apparatus, is packaged into secretory vesicles, and is transported to the plasma membrane for release. Thus, differential subunit processing, glycosylation, heterodimer assembly, and intracellular trafficking together provide important post‐translational checkpoints regulating IL‐12 secretion.

Although both the p35 and p40 subunits must be co‐expressed to form bioactive IL‐12, their production is independently regulated as discussed above. Coexpression with p40 markedly increases steady‐state levels of p35, indicating that p40 stabilizes and promotes p35 production. Using HEK293 cells that were transfected with plasmids encoding the IL‐12 subunits alone or together, it was shown that the p40 subunit plays a critical role in promoting the stability, intracellular trafficking, and secretion of the p35 subunit [63]. Moreover, p40's secretion is independent of glycosylation, whereas p35 secretion strictly requires glycosylation, as blocking this process with Tunicamycin prevents its release [65].

## 6 Polarized IL‐12 Release

DCs activate T cells by forming a so‐called immunological synapse, a tight interface between the DC and the T cell where antigen presentation occurs. Activation of naive T cells requires two signals that act during formation of the immunological synapse. Signal 1 consists of antigen‐specific recognition, in which the T cell receptor binds to a peptide‐major histocompatibility complex (pMHC) displayed on the DC surface. Signal 2 provides costimulation, most notably through engagement of CD28 on the T cell with B7‐1 (CD80) or B7‐2 (CD86) on the DC. Signal 1 alone is insufficient and typically results in T cell anergy, whereas the combination of signal 1 and signal 2 promotes full activation, including IL‐2 production, clonal proliferation, and survival. However, the differentiation into effector T cells also requires cytokines, called Signal III, such as IL‐12 for Th1 differentiation [66].

To facilitate cytokine release, DCs engaged in the immunological synapse undergo dynamic remodeling of their microtubule cytoskeleton, characterized by reorientation of the microtubule‐organizing center (MTOC) toward the interacting T cell, thereby facilitating polarized secretion of IL‐12 and efficient T cell activation. Newly synthesized IL‐12 locates around the MTOC at early stages after TLR ligation, and relocalizes at the interface between the DC and T cell in antigen‐specific synapses [67]. Thereby, IL‐12 release can be highly directed toward the immunological synapse to promote effective cell–cell communication, and this likely allows for a more targeted immune response as it prevents or limits bystander activation of nearby T cells that do not carry the cognate T cell receptor [68, 69]. The small Rho‐GTPase Cdc42 is a key regulator of this polarization process in DCs. It is recruited to the DC‐T cell interface and is essential for proper positioning of the MTOC and the associated Golgi apparatus toward the interacting T cell. Blocking this cytoskeletal polarization by Cdc42 silencing or treatment with the microtubular inhibitor colchicine does not impair immunological synapse formation but disrupts the polarized delivery of IL‐12 to the interacting T cell. As a result, IFN‐γ production and T cell survival are reduced [67].

Polarized IL‐12 trafficking in DC is not unique for synapses with T cells, because it also occurs in synapses with NK cells. Confocal imaging revealed that within 25 min of DC contact with NK cells, IL‐12 redistributes from distal cytoplasmic granules toward the DC‐NK synapse in ~78% of DCs [70]. This polarization requires an intact cytoskeleton and the presence of lipid rafts, microdomains in cell membranes enriched in cholesterol and sphingolipids. Incubation of DCs with inhibitors of microtubules (nocodazole) or actin polymerization (cytochalasin B), dispersing the lipid rafts by cholesterol extraction with methyl‐β‐cyclodextrin, as well as WASP deficiencies, disrupted IL‐12 localization to the synapse with NK cells [70].

## 7 IL‐12 Trafficking via Late Endosomes

IL‐12 is not released directly from the Golgi network at the plasma membrane but traffics via an unconventional route regulated by the soluble N‐ethylmaleimide‐sensitive‐factor attachment protein receptor (SNARE) protein vesicle‐associated membrane protein 7 (VAMP7), which is essential for clustering and targeted secretion of IL‐12 at the immunological synapse. As mentioned above, newly synthesized IL‐12 is transported in DCs along microtubules to the immunological synapse, and this process depends on the SNARE protein VAMP7. Secretion of the bioactive IL‐12 dimer is about 57% reduced in VAMP7 knockout mice, whereas p40 secretion and IL‐6 secretion were mostly unaffected. Furthermore, deletion of VAMP7 impaired the differentiation of T cells into IFN‐γ‐producing cells, indicating that VAMP7‐dependent IL‐12 trafficking in DCs is essential for effective T cell effector differentiation [71].

Evidence from other cell types suggests that VAMP7‐dependent secretion can be regulated by mechanical cues. In COS7 cells, substrate rigidity modulates VAMP7‐mediated exocytosis by controlling the peripheral distribution of VAMP7‐positive vesicles through the opposing actions of the Longin domain to leucine‐rich repeat kinase 1 (LRRK1) and the kinesin‐associated protein VARP [72]. This mechanosensitive regulation may be relevant to DCs, as formation of the immunological synapse involves extensive cytoskeletal remodeling and mechanically active interactions between DCs and T cells [67, 68]. Thus, mechanical forces generated during synapse formation could potentially influence the positioning and availability of VAMP7‐positive IL‐12‐containing compartments for polarized secretion. Whether this mechanism operates in DCs, however, remains to be established.

IL‐12 production in DCs also depends on Galectin‐9 (Gal‐9), a ß‐galactoside‐binding protein. Gal‐9 interacts with vesicle‐associated membrane protein 3 (VAMP3), a SNARE protein localized in the recycling and early endosomal compartments. Like VAMP7, VAMP3 regulates cytokine secretion in DCs by governing vesicular trafficking from the Golgi apparatus to the plasma membrane [73]. In the absence of Gal‐9, VAMP3‐positive vesicles fail to translocate efficiently to the plasma membrane, resulting in impaired IL‐12 release. However, this mechanism is not specific for IL‐12, as production of IL‐6, TNF, and IL‐10 was also impaired [74]. It remains unclear whether the R‐SNAREs VAMP3 and VAMP7 function sequentially or redundantly in IL‐12 release. Both SNAREs might form a complex with the Q‐SNARE syntaxin‐3 (STX3) [73], as STX3 is also responsible for secretion of IL‐12, as found by microarray screening. siRNA‐mediated depletion of STX3 in neutrophil‐like dHL‐60 cells impaired the release of p40, IL‐1α, IL‐1β, CCL4, and Matrix Metalloproteinase‐9 (MMP‐9), without affecting other cytokines [75].

## 8 IL‐12 in Pathology

Given its central role in linking innate and adaptive immunity, dysregulation of IL‐12 can have markedly different pathological consequences. As mentioned above, IL‐12 is essential for protection against many intracellular pathogens and contributes to antitumor immune responses by promoting IFN‐γ production and type 1 immunity [76, 77]. Conversely, excessive or sustained activation of the IL‐12/Th1 axis can contribute to chronic inflammatory and immune‐mediated pathology, although some effects historically attributed to IL‐12 are now known to involve the closely related IL‐23 pathway [78, 79]. Thus, both insufficient and dysregulated IL‐12 activity can be detrimental, highlighting the need for tight control of its production and signaling across infectious, inflammatory, autoimmune, and neoplastic diseases (Figure 5).

**FIGURE 5:**
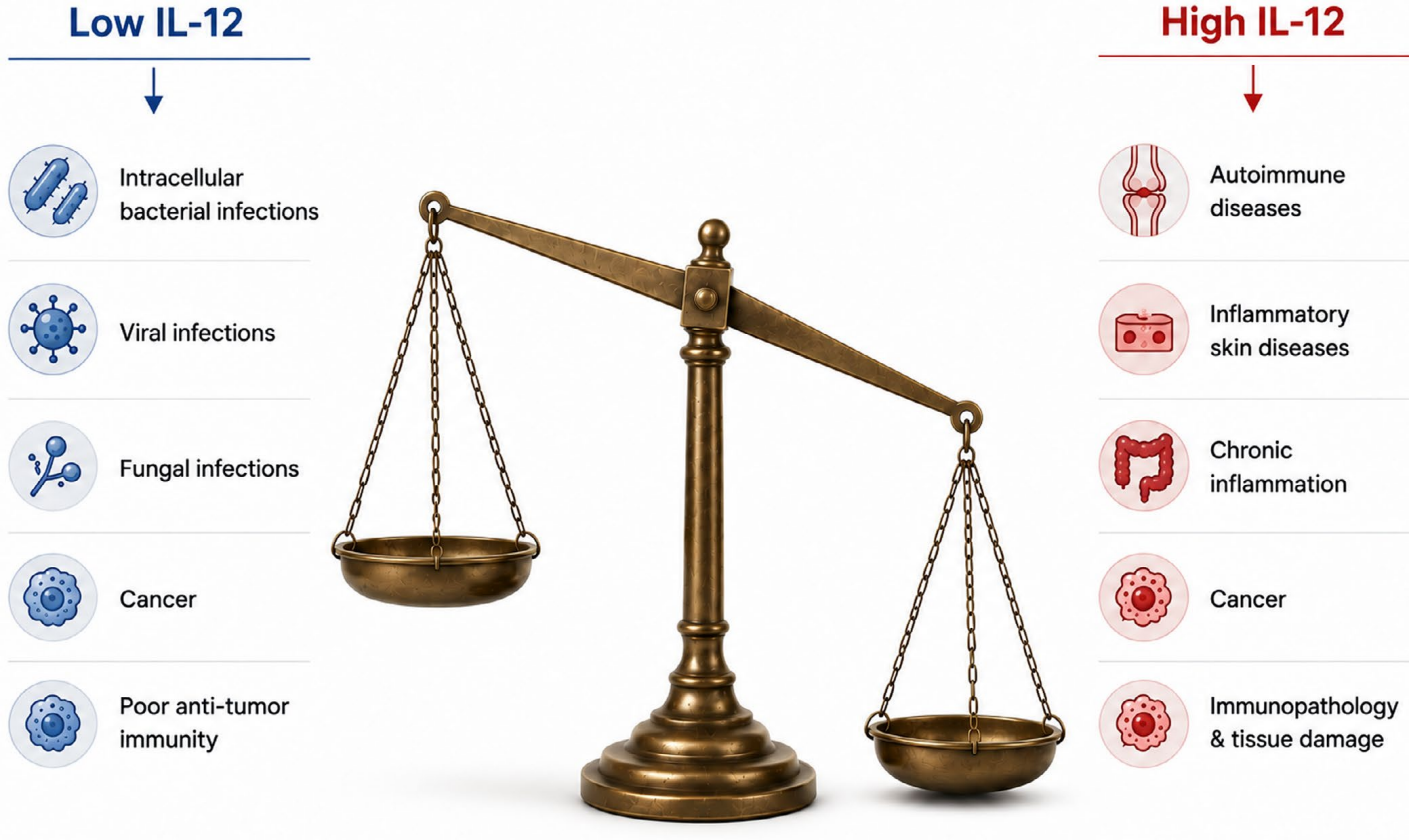
Graphical summary of IL‐12‐related pathologies. The figure illustrates the importance of tightly regulated IL‐12 production and signaling for immune homeostasis. Low IL‐12 activity is associated with impaired host defense and weak antitumor immunity, whereas excessive or dysregulated IL‐12 signaling can contribute to inflammatory, autoimmune, and tissue‐damaging pathology. Thus, tight regulation of IL‐12 production and signaling is essential for immune homeostasis across multiple disease settings.

## 9 Parasite and Bacterial Infections: Regulation of IL‐12

Given the central role of IL‐12 in immune responses, it is no surprise that intracellular pathogens manipulate IL‐12 responses to evade immunity. Pathogens employ multiple strategies to evade host immunity by targeting IL‐12 production and signaling. Some, such as *Toxoplasma gondii* and *Leishmania major*, directly suppress IL‐12 synthesis in macrophages and DCs by modulating the NF‐κB pathway [80, 81, 82]. Some pathogens, for example, *M. tuberculosis* , induce host anti‐inflammatory cytokines, including IL‐10 and TGF‐β, which indirectly inhibit IL‐12 expression [83, 84]. Administration of IL‐12 in tuberculosis infections enhances bacterial clearance, likely through induction of IFN‐γ because direct IFN‐γ administration also exerts this effect [85, 86]. In contrast, *Salmonella* employs the opposite strategy and increases IL‐12 signaling as a virulence strategy, as its infection triggers excessive IL‐12 production that elevates expression of the transcription factor T‐bet in T cells, suppressing T follicular helper (Tfh) cell differentiation and compromising the adaptive immune response [87].

The mechanisms by which pathogens suppress IL‐12 production differ considerably between pathogens. For example, *Leishmania donovani* modulates small G‐protein signaling in infected macrophages, particularly affecting the Ras family. Infection increases N‐Ras expression and activity, while suppressing K‐Ras and H‐Ras. Active N‐Ras promotes ERK1/2 phosphorylation and reduces p38 phosphorylation, which favors IL‐10 and suppresses IL‐12 production. Pharmacological inhibition of Ras using farnesylthiosalicylic acid reverses this effect, decreasing IL‐10 and increasing IL‐12 expression, thereby enhancing macrophage proinflammatory responses [88].

*M. tuberculosis* has evolved multiple strategies to suppress IL‐12 production and thereby weaken protective Th1 immunity. The cell wall component mannose‐capped lipoarabinomannan (ManLAM) acts as an anti‐inflammatory molecule that inhibits IL‐12 and TNF production, while promoting IL‐10 secretion in macrophages and DCs [89]. The *mmaA4* gene (also known as *hma* or *Rv0642c*) in *M. tuberculosis* encodes the enzyme hydroxymycolate synthase MmaA4, which is crucial for modifying cell wall mycolic acids. It is essential for synthesizing methoxy‐ and keto‐mycolates, playing a key role in bacterial virulence and immune evasion by suppressing host p40 production [90, 91]. The bacterium also induces negative regulatory molecules such as IRAK‐M, a suppressor of TLR signaling, which disrupts IRAK‐1–TRAF6 interactions and blocks downstream pathways required for IL‐12 production [92]. Certain strains additionally suppress IL‐12 production through an epigenetic mechanism. In macrophages infected with live, virulent *M. tuberculosis* , histone deacetylases (HDACs) are recruited to the *IL12B* promoter, resulting in hypoacetylation of histone H3, thereby suppressing expression [93].

Immune clearance of the parasitic alveolate *T. gondii* also relies on IL‐12 signaling. Following *T. gondii* infection in mice, DCs and macrophages act as primary sensors, detecting parasite‐derived profilin and GPI‐anchored proteins via TLR11 and TLR12 to induce rapid, high‐level production of IL‐12 [94]. Deficiencies in IL‐12 are strongly linked to uncontrolled parasite replication and high susceptibility to *T. gondii* infection [95]. A more recent study found that CD8α^+^ DCs are the main source of IL‐12 during *T. gondii* infection. Batf3^−^/^−^ mice, which lack CD8α^+^ DCs, showed reduced IL‐12 and IFN‐γ production and are highly susceptible to infection. Intraperitoneal administration of IL‐12 restores resistance in these mice [96].

Since IL‐12 plays key roles in immune defenses against bacterial pathogens, IL‐12 supplementation is a therapeutic strategy. In the past century, many studies aimed to increase immune responses by IL‐12 administration for a wide range of pathologies, including cancer, infectious disease, airway inflammation, and as a vaccine adjuvant [97, 98, 99, 100, 101]. For example, IL‐12 gene therapy can protect mice from lethal *K. pneumoniae* infections. CBA/J mice were intratracheally challenged with *K. pneumoniae* , resulting in time‐dependent induction of *Il12a* and *Il12b* mRNA and IL‐12 protein expression in the lung. Functional blockade of IL‐12 through intraperitoneal administration of neutralizing anti‐IL‐12 serum at the time of bacterial inoculation led to a 12‐fold increase in pulmonary bacterial burden at 48 h compared with control serum‐treated animals, along with significantly reduced short‐ and long‐term survival. To evaluate the therapeutic potential of localized IL‐12 overexpression, mice received intratracheal delivery of a nonreplicating adenoviral vector encoding *Il12a* and *Il12b*, thereby delivering IL‐12 to the lung in mice with *K. pneumoniae* infection. This conferred a 45% long‐term survival rate in infected animals, whereas all mice receiving control adenovirus succumbed to infection, highlighting the critical protective role of IL‐12 in host defense against *K. pneumoniae* . Therefore, localized, transient cytokine delivery via intratracheal adenoviral vectors represents both a valuable experimental approach and a promising therapeutic strategy [102].

## 10 Therapeutic Targeting of IL‐12 in Cancer

IL‐12 can serve as a therapeutic target in cancer (Table 1). For example, IL‐12 administration was shown to inhibit tumor growth and improve the tumor microenvironment in multiple preclinical models [103]. In humans, it has been reported that intratumoral injection of an adenovirus encoding human IL‐12 (AFIL‐12) in 21 patients with advanced gastrointestinal malignancies was well tolerated with one partial response and six cases of stable disease [104]. Biological activity was evidenced by increased serum IFN‐γ levels and enhanced lymphocytic infiltration in four of the 10 evaluated patients. In other human clinical trials, intratumoral delivery of Tavokinogene telseplasmid (TAVO, a DNA‐based IL‐12 plasmid) in patients with advanced or metastatic triple‐negative breast cancer showed safety, enhanced CD8^+^ T cell infiltration, and antigen presentation, which are required for antitumor efficacy [105].

**TABLE 1:** Current therapeutic strategies targeting. The table summarizes established IL‐12/IL‐23‐targeting therapies, discontinued or experimental approaches, and emerging IL‐12‐based treatments currently under clinical investigation from https://clinicaltrials.gov.

Strategy/agent	IL‐12 mechanism	Pathology	Development/status as of Aug 2026	Key reference/trial
Ustekinumab	Monoclonal antibody against the shared p40 subunit, thereby blocking both IL‐12 and IL‐23 signaling	Plaque psoriasis, psoriatic arthritis, Crohn's disease, ulcerative colitis	Approved	FDA prescribing information; pivotal clinical studies
Apilimod (STA‐5326)	PIKfyve inhibitor that suppresses TLR‐dependent IL‐12/IL‐23 expression	Crohn's disease, rheumatoid arthritis; historically psoriasis	Phase II development for inflammatory disease unsuccessful/discontinued	Sands et al. 2010; Krausz et al. 2012; Cai et al. 2013
Selective IL‐12‐pathway inhibition	Genetic disruption of IL‐12/IL‐12R signaling	Alzheimer's disease	Preclinical	Schneeberger et al. 2025
XTX301	Systemically administered tumor‐activated IL‐12, designed to remain largely inactive outside the tumor microenvironment	Advanced solid tumors, including HNSCC, melanoma, NSCLC, ovarian, prostate cancer and TNBC	Phase I/II, recruiting	NCT05684965
Tolododekin alfa (ANK‐101)	Intratumorally anchored IL‐12 designed for prolonged local retention and reduced systemic exposure	Advanced/metastatic NSCLC	Phase Ib, recruiting, in combination with PD‐1/PD‐L1 blockade	NCT07027514; Park et al., 2025
PDS01ADC (formerly NHS‐IL12/M9241)	Tumor‐targeted IL‐12 immunocytokine designed to concentrate IL‐12 within necrotic tumor regions	Prostate cancer and other advanced solid tumors	Multiple Phase I/II trials	For example, NCT04633252, NCT05286814, NCT06096870
T3011	Oncolytic HSV engineered to locally express IL‐12 and an anti‐PD‐1 antibody	Advanced solid tumors	Phase Ib/IIa, recruiting	NCT06214143
attIL12‐T cells	Autologous T cells engineered to express membrane‐anchored, tumor‐targeted IL‐12, restricting cytokine activity to the tumor/T cell interface	Advanced or metastatic soft‐tissue and bone sarcomas, including osteosarcoma	Phase I, recruiting	NCT05621668
IMNN‐001 (GEN‐1), OVATION‐3	Intraperitoneal IL‐12 plasmid formulated with a PEG–PEI–cholesterol delivery system plus chemotherapy	Advanced epithelial ovarian, fallopian‐tube or primary peritoneal cancer	Phase III, recruiting	NCT06915025
IMNN‐001 + bevacizumab + chemotherapy	Local IL‐12 plasmid gene therapy combined with antiangiogenic therapy and chemotherapy	Advanced ovarian, fallopian‐tube or primary peritoneal cancer	Phase I/II, recruiting	NCT05739981
STX‐001 ± pembrolizumab	Intratumoral lipid nanoparticle containing self‐replicating RNA encoding IL‐12	Advanced solid tumors, including melanoma	Phase I/II, recruiting	NCT06249048
VLPONC‐01 ± pembrolizumab	Intratumoral viral replicon particles carrying self‐amplifying RNA encoding IL‐12	Head and neck squamous‐cell carcinoma	Phase I, recruiting	NCT06736379
T3011 + PD‐1/PD‐L1 blockade	Oncolytic HSV engineered to locally express IL‐12 and an anti‐PD‐1 antibody	Advanced solid tumors	Phase Ib/IIa, recruiting	NCT06214143
M032 + pembrolizumab	Oncolytic HSV‐1 engineered to express human IL‐12, combined with PD‐1 blockade	Newly diagnosed or recurrent/progressive high‐grade malignant glioma	Phase I/II, recruiting	NCT05084430
M032	Intratumoral engineered HSV‐1 expressing human IL‐12	Newly diagnosed diffuse midline glioma after radiotherapy	Phase I, recruiting/enrolling	NCT07076498
Tolododekin alfa (ANK‐101) + anti‐PD‐1/PD‐L1	Intratumorally anchored IL‐12 designed for prolonged tumor retention and reduced systemic exposure	Advanced/metastatic NSCLC	Phase Ib, recruiting	NCT07027514
PDS01ADC + docetaxel ± abiraterone	Tumor‐targeted IL‐12 immunocytokine	Metastatic prostate cancer	Phase I/II, recruiting	NCT04633252
PDS01ADC + liver‐directed/systemic therapy	Tumor‐targeted IL‐12 immunocytokine combined with liver‐directed chemotherapy	Liver‐dominant metastatic colorectal cancer, intrahepatic cholangiocarcinoma or adrenocortical carcinoma	Phase II, recruiting	NCT05286814
PDS01ADC + enzalutamide	Tumor‐targeted IL‐12 immunocytokine combined with androgen‐receptor blockade	Recurrent prostate cancer	Phase II, recruiting	NCT06096870

Abbreviations: HNSCC, head and neck squamous‐cell carcinoma; HSV, herpes simplex virus; IL‐12, interleukin‐12; IL‐23, interleukin‐23; NSCLC, non‐small‐cell lung cancer; PD‐1, programmed cell death protein 1; PD‐L1, programmed death‐ligand 1; PEG, polyethylene glycol; PEI, polyethyleneimine; PIKfyve, phosphoinositide kinase, FYVE‐type zinc finger containing; RNA, ribonucleic acid; TLR, toll‐like receptor; TNBC, triple‐negative breast cancer.

However, while IL‐12 is highly effective in stimulating antitumor immunity, its systemic administration can lead to toxicity [106, 107]. IL‐12 is a pleiotropic cytokine and systemic administration induces rapid increases in proinflammatory cytokines, including IFN‐γ, TNF, and IL‐6 [108]. Therefore, early clinical trials involving systemic administration of recombinant human IL‐12 (rhIL‐12) were limited by significant toxicities associated with excessive induction of proinflammatory cytokines. The most commonly reported adverse events included fever and flu‐like symptoms, along with fatigue, nausea, vomiting, diarrhea, and headache. Hepatotoxicity was also frequently observed, as indicated by elevated alanine aminotransferase (ALT) and aspartate aminotransferase (AST) levels. In addition, hematologic toxicities such as leukopenia, anemia, neutropenia, and thrombocytopenia were commonly reported [109]. In addition to toxicity, the therapeutic potential of systemic IL‐12 administration is limited by a narrow therapeutic window, and off‐target effects due to lack of tumor specificity [110, 111].

To overcome this systemic toxicity, strategies that locally deliver IL‐12 to the tumor or infection site have been developed [110]. For example, DCs have been engineered to stably express IL‐12 via adenoviral or retroviral vectors. These modified DCs migrate normally to the lymph nodes and can improve tumor suppression and survival [112]. This strategy has been tested in a Phase I trial, where autologous DCs were transfected to express IL‐12 and were administered intratumorally to patients with metastatic gastrointestinal carcinomas. Among 17 treated patients, one achieved a partial response and two exhibited stable disease. Serum IFN‐γ, but not IL‐12, levels were elevated post‐treatment. Four transient cases of grade 3 lymphopenia were observed, with no grade 4 toxicities reported. In vitro IL‐12 production was highly variable, likely reflecting differences in DC susceptibility to adenoviral infection [113].

Combination strategies have been extensively explored to enhance the antitumor activity of IL‐12. By activating complementary immune pathways, combinations may increase therapeutic efficacy without requiring further escalation of systemic IL‐12 exposure. Consequently, the field has increasingly shifted toward strategies that combine spatially restricted IL‐12 activity with complementary immunotherapies, particularly immune‐checkpoint blockade, adoptive cell therapy, and engineered delivery platforms [103, 114, 115].

A particularly well‐developed approach combines local IL‐12 delivery with programmed cell death protein 1/programmed death‐ligand 1 (PD‐1/PD‐L1) blockade. This strategy is mechanistically attractive because IL‐12 promotes IFN‐γ production, cytotoxic T cell and NK cell activity, and immune cell recruitment into the tumor microenvironment, while the resulting inflammatory response can increase checkpoint‐ligand expression and thereby create a rationale for simultaneous checkpoint inhibition. In patients with poorly immunogenic advanced melanoma, intratumoral electroporation of the IL‐12‐encoding TAVO plasmid combined with pembrolizumab achieved a 41% objective response rate and was associated with increased intratumoral immune cell infiltration and activation of an IL‐12/IFN‐γ inflammatory program [116]. More recently, neoadjuvant TAVO combined with nivolumab in operable locoregionally advanced melanoma resulted in a 60% pathological complete response and an 80% major pathological response among 15 patients undergoing surgery, accompanied by increased CD8^+^ T cell infiltration and immune activation [117]. Nevertheless, clinical efficacy remains context‐dependent. The tumor‐targeted IL‐12 immunocytokine M9241 (NHS‐IL12) combined with the anti‐PD‐L1 antibody avelumab was generally tolerable and produced prolonged complete responses in individual patients during dose escalation, but no objective responses were observed in the subsequent urothelial carcinoma expansion cohort [118].

In parallel, substantial effort has focused on engineering IL‐12 delivery itself. Nucleic‐acid‐based approaches provide transient or sustained IL‐12 production within tumors, while avoiding repeated systemic administration of recombinant cytokine. A self‐replicating RNA‐lipid nanoparticle formulation encoding IL‐12 produced preferential tumor‐associated cytokine expression, remodeled the tumor microenvironment and enhanced anti‐PD‐1 activity in preclinical models, with encouraging tolerability in mice and non‐human primates [119]. Similarly, tumor‐tailored ionizable lipid nanoparticles carrying IL‐12 circular RNA induced sustained local IL‐12 expression, increased CD8^+^ T cell infiltration and promoted tumor regression in experimental mouse lung cancer models [120]. A conceptually related strategy is anchored IL‐12 linked to the aluminum hydroxide adjuvant, by which IL‐12 is retained within the injected tumor. In a first‐in‐human phase I trial, tolododekin alfa, anchored IL‐12, showed prolonged local retention with limited systemic exposure, increased intratumoral CD8^+^ T cells and inflammatory gene expression, and produced no dose‐limiting toxicities or treatment‐related serious adverse events in the initial cohort [121].

Viral and cellular platforms provide additional means of coupling tumor targeting with localized IL‐12 delivery. T3011, an intratumorally administered oncolytic herpes simplex virus engineered to express both IL‐12 and an anti‐PD‐1 antibody, demonstrated a favorable overall safety profile together with increased CD8^+^ T cell infiltration and evidence of tumor immune remodeling in a I/IIa study of advanced solid tumors [122].

IL‐12 can also be used to improve chimeric antigen receptor (CAR)‐T cell therapy. Compared to CAR‐T cells given alongside exogenous PD‐L1 antibodies and recombinant IL‐12, this approach showed improved immune activity in the tumor while minimizing side effects in preclinical solid tumor models, including murine prostate cancer and ovarian cancer models [123]. Another study engineered CAR‐T cells with improved efficacy against solid tumors and lower toxicity by incorporating a membrane‐bound IL‐12 into the CAR‐T cells. This was achieved by linking the p35 and p40 subunits to a membrane‐anchoring CD28 transmembrane domain [124]. Preclinical studies show that these CAR‐T cells induce IL‐12‐dependent IFN‐γ and induce potent ovarian cancer immune responses [124].

Similarly, increased expression of IL‐12 is also a strategy to improve adoptive T cell therapy. To reduce IL‐12‐mediated off‐tumor toxicity, researchers identified a linear peptide that targets cell‐surface vimentin (CSV), a marker broadly expressed across tumor types and associated with aggressive, mesenchymal‐like cancer cells. Based on this approach, T cells were engineered to express cell membrane‐anchored and tumor‐targeted IL‐12 (attIL12‐T cells), in which IL‐12 is retained on the T cell surface and directed toward CSV^+^ tumor cells. T cells were engineered to express membrane‐anchored and tumor‐targeted IL‐12, and these were administered to murine osteosarcoma and melanoma models. Infusion of the engineered T cells robustly inhibited tumor growth, selectively targeting CSV^+^ tumor cells, increasing intratumoral IFN‐γ, promoting DC maturation, secondary T cell activation, and antigen spreading [125].

Finally, the toxicity of IL‐12 administration can be prevented or limited by masking IL‐12 by fusing it to a tumor‐activated protease‐cleavable domain. This mask is attached via a linker that is cleaved by matrix metalloproteinases (like MMP2, MMP9), urokinase‐plasminogen activators (uPA), and serine proteases (SP) that are overexpressed in the tumor microenvironment. In mouse models of adenocarcinoma and melanoma, masked IL‐12 could enhance responses to checkpoint inhibitors and avoid systemic toxicities while remaining selectively activatable in patient tumor lysates [126]. Furthermore, XTX301, a tumor‐activated IL‐12 fusion protein linked to a human Fc domain through a protease‐cleavable linker and rendered pharmacologically inactive by an IL‐12 receptor β2 masking domain, was activated specifically in the tumor microenvironment by MMP cleavage. This tumor‐restricted activation promoted CD8^+^ T cell infiltration and proinflammatory signaling, inhibiting tumor growth while minimizing systemic toxicity, as shown in preclinical mouse and primate studies [127]. Targeted delivery approaches are limited by incomplete tumor penetration, potential immunogenicity of the fusion protein, and off‐target tissue binding [114].

Together, these studies illustrate a broader transition in IL‐12‐based cancer therapy: rather than simply combining systemically administered IL‐12 with additional immune stimulants, current approaches increasingly seek to localize or condition IL‐12 activity within the tumor and couple it to therapies that exploit the inflammatory tumor microenvironment generated by IL‐12. These strategies developed in oncology to mitigate the toxicity of IL‐12 administration may be translatable to therapeutic approaches for infectious diseases, enabling effective immune activation while minimizing systemic adverse effects [103, 114, 115].

## 11 Therapeutic Targeting of IL‐12 in Autoimmune and Metabolic Disease

IL‐12 cytokine is elevated in several autoimmune diseases, particularly those driven by Th1‐mediated immune responses. Whereas IL‐12 is essential for immune defenses against microbial pathogens and cancer, excessive IL‐12 production is a hallmark of these diseases. Conditions associated with abnormal IL‐12 levels include psoriasis, multiple sclerosis, rheumatoid arthritis, systemic lupus erythematosus, inflammatory bowel disease, systemic sclerosis, primary Sjögren's syndrome (pSS), autoimmune nephropathy, Crohn's disease, and type 1 and type 2 diabetes mellitus [128, 129]. For example, IL‐12 contributes to the pathogenesis of pSS, a condition marked by chronic B‐cell activation and an increased risk of lymphoma, particularly non‐Hodgkin lymphoma. Not only are serum IL‐12 levels elevated in patients with active disease, but polymorphisms in *IL12A* have also been linked to pSS [130].

It has also been shown that IL‐12 contributes to type I diabetes. *Il12b* mRNA is upregulated in the pancreas and spleen of cyclophosphamide‐treated non‐obese diabetic (NOD) mice and diabetes‐prone BioBreeding (BB) rats before and during disease onset [131]. Administration of recombinant IL‐12 accelerated diabetes in NOD mice, accompanied by increased Th1 cytokine production by islet‐infiltrating T cells [132]. Treatment with the IL‐12 antagonist p40_2_ did not trigger the typical proinflammatory activity associated with the IL‐12 heterodimer. When administered before the onset of insulitis, it shifted pancreatic CD4^+^ T cells toward a Th2 phenotype and reduced both spontaneous and accelerated diabetes [133]. In contrast, treatment when insulitis was already established had limited effects in reducing pancreas‐infiltrating Th1 cells and did not affect Th2 cell differentiation [133]. These findings indicate that IL‐12 is critical in early type I diabetes pathogenesis in NOD mice, while Th2 induction within pancreatic infiltrates is protective [131, 132, 133].

More recent studies in NOD mice have shown that inhibition of IL‐12‐induced Th1 differentiation with sorafenib, an FDA‐approved tyrosine kinase inhibitor, reduced pancreatic Th1‐cell accumulation, ameliorated insulitis, and decreased diabetes development. Mechanistically, sorafenib inhibited IL‐12‐induced phosphorylation of JAK2 and STAT4, further supporting the involvement of the IL‐12/JAK2/STAT4 axis in diabetogenic Th1 responses [134]. In patients with type 1 diabetes, circulating p40 levels were increased compared with individuals with normal glucose tolerance, while higher IL‐12 concentrations were associated with poorer glycemic time‐in‐range [135].

Altered IL‐12 signaling has also been implicated in chronic inflammatory diseases outside classical autoimmunity. For example, IL‐12 not only plays a role in type I but also in type II diabetes [136]. Type 2 diabetes was induced by feeding wild‐type, p40 knockout, and p35 knockout mice a high‐fat diet (HFD) for 12 weeks, after which body weight, glucose tolerance, and insulin sensitivity were evaluated. IL‐12 deficiency enhanced angiogenesis and improved blood flow recovery in obese diabetic mice, likely through mechanisms involving endothelial nitric oxide synthase (eNOS), Akt (a serine/threonine‐specific protein kinase) signaling, vascular endothelial growth factor receptor 2 (VEGFR2), and modulation of oxidative stress and inflammation [137].

In asthma, genetic variation within *IL12B* has been associated with disease severity [138]. Recent studies support a context‐dependent role for IL‐12 in asthma, particularly in regulating the balance between Th1‐ and Th2‐polarized immunity. In an experimental mouse model of allergic asthma, where the animals are exposed to dust mite allergens to trigger airway inflammation, reduced IL‐12 production by airway DCs was associated with epigenetic suppression of *Irf5* and enhanced Th2 polarization. Inhibition of the methyltransferase‐like protein‐5 (METTL5), a methyltransferase involved in N6‐methyladenosine (m6A), restored this pathway and attenuated allergic inflammation [139]. More recently in the same mouse dust mite model, DNA (cytosine‐5)‐methyltransferase 1 (DNMT1)‐dependent hypermethylation of the *Il12b* promoter was shown to suppress p40 expression in DCs during allergen sensitization. Pharmacological or DC‐specific inhibition of DNMT1 restored *Il12b* expression and reduced airway eosinophilia and Th2 cytokine production, further supporting a protective role for IL‐12‐associated signaling in allergic airway inflammation [140].

However, IL‐12 also exerts opposite effects, as it can promote asthma development. In a mouse model of asthma, IL‐12 was found to inhibit Runt‐related transcription factor 3 (RUNX3) levels and increase the hypoxia‐inducible factor 1‐alpha (HIF‐1α) and the NLR family pyrin domain containing 3 (NLRP3) expression. In heterozygous Runx3 knockout mice, serum IgE, bronchoalveolar lavage fluid cytokines, and lung levels of IL‐12, HIF‐1α, and NLRP3 were markedly increased. Human bronchial epithelial BEAS‐2B cells were also treated with varying doses of IL‐12, and expression of Runx3, HIF‐1α, and NLRP3 was evaluated by Western blot and immunofluorescence. It was shown that higher IL‐12 concentrations led to decreased RUNX3. Under normal conditions, RUNX3 acts as a protective factor by inhibiting the HIF‐1α/NLRP3 signaling pathway. When RUNX3 is downregulated (as seen in asthma models), this inhibition is lost, leading to increased lung epithelial cell injury. These results indicate that IL‐12 contributes to asthma development by modulating the HIF‐1α/NLRP3 pathway via Runx3 [141].

Son et al. analyzed 221 sputum samples from 143 patients with severe asthma to investigate whether autoantibodies against the macrophage scavenger receptor with collagenous structure (MARCO) contribute to recurrent airway infections. Anti‐MARCO IgG was detected in 36% of patients and was particularly associated with mixed eosinophilic‐neutrophilic airway inflammation and an increased risk of infective exacerbations. Functionally, anti‐MARCO‐containing immunoglobulins impaired bacterial uptake by macrophages in vitro. Interestingly, sputum IL‐12, together with IL‐13 and IL‐15, was independently associated with the anti‐MARCO‐positive phenotype, indicating that these infection‐prone severe‐asthma variations are characterized by overlapping type 1 and type 2 inflammatory signals [142]. Furthermore, a recent prospective study of patients with severe eosinophilic asthma reported a modest increase in circulating IL‐12 following treatment with currently approved monoclonal antibodies for severe asthma treatment such as benralizumab (anti‐IL‐5 receptor), dupilumab (anti‐IL‐5), or mepolizumab (anti‐IL‐4 receptor), although IL‐12 concentrations were low and the clinical significance of this change remains unclear [143].

Together, these findings suggest that IL‐12 cannot be considered uniformly deficient or protective in asthma; rather, its regulation depends on the inflammatory conditions, cellular source, and disease context.

## 12 Therapeutic IL‐12 Inhibition

IL‐12 inhibition is a well‐established therapeutic strategy for the treatment of autoimmune diseases (Table 1). Older studies showed that intraperitoneal administration of monoclonal anti‐IL‐12 antibodies in murine models of chronic intestinal inflammation can suppress inflammation [144]. Similarly, the addition of anti‐IL‐12 antibodies to lymph node cell cultures also decreased IFN‐γ secretion [145]. These findings have been translated to the clinic and IL‐12 targeting antibodies are now widely used. For example, ustekinumab is a human monoclonal antibody that targets the p40 subunit shared by IL‐12 and IL‐23 [146]. It is used to treat inflammatory conditions like psoriasis, psoriatic arthritis, Crohn's disease, and ulcerative colitis by suppressing both IL‐12‐driven Th1 and IL‐23‐driven Th17 pathways [147, 148].

IL‐12 production has also been targeted at the level of organelle trafficking. STA‐5326, also known as apilimod, is an orally active small‐molecule inhibitor that suppresses transcription of *IL12A* and *IL12B*, reducing production of both IL‐12 and IL‐23. Apilimod targets the lipid kinase PIKfyve which is responsible for the maturation of early into late endosomes, thereby disrupting late endosomal and lysosomal functions [149]. Apilimod likely suppresses IL‐12 production as IL‐12 is secreted via compartments of late endosomal or lysosomal nature as described above [71]. In mice, oral apilimod selectively inhibited Th1 responses, decreased IFN‐γ production, and ameliorated inflammation in a Severe Combined Immunodeficiency (SCID) model of inflammatory bowel disease [150]. However, apilimod failed to meet primary endpoints in Phase 2 trials for Crohn's disease and rheumatoid arthritis, leading to the discontinuation of its development for these indications [151, 152].

IL‐12 inhibitors might not only be useful for the treatment of autoimmune diseases but are also potential treatments for neuroendocrine diseases. A recent study demonstrated that genetic ablation of IL‐12 signaling in a mouse model of Alzheimer's disease restored mature oligodendrocyte numbers, improved myelin homeostasis, rescued the amyloid‐β‐dependent loss of parvalbumin‐positive interneurons, and normalized phagocytosis‐related alterations in microglia. Moreover, IL‐12 protein levels were elevated in human Alzheimer's disease brains compared to age‐matched controls, and human oligodendrocyte‐like cells showed strong responsiveness to IL‐12 stimulation [153].

## 13 Conclusion

The above review includes IL‐12 as a pivotal cytokine produced primarily by antigen‐presenting cells that links innate and adaptive immunity by inducing IFN‐γ production and promoting Th1 cell differentiation. Its biological activity relies on the tightly regulated, yet independent, synthesis and post‐translational processing of its p35 and p40 subunits, as well as specialized intracellular trafficking and polarized secretion at immune synapses involving cytoskeletal remodeling and SNARE proteins such as VAMP7 and STX3. IL‐12 is essential for protective immunity against intracellular pathogens, and disruptions in IL‐12 signaling result in heightened susceptibility to infection, while many pathogens actively suppress IL‐12 to evade host defenses. Inhibition of IL‐12 signaling is a key strategy for treatment of autoimmune diseases, whereas increasing IL‐12 has gained prominence for treatment of infections and cancer, where innovative delivery approaches, including tumor‐restricted, tethered, and cell‐based IL‐12 strategies seek to exploit its potent antitumor properties while limiting systemic toxicity.

## Author Contributions

**Anthi Psoma:** conceptualization, writing – original draft. **Geert van den Bogaart:** resources, writing – review and editing, supervision.

## Ethics Statement

The authors have nothing to report.

## Conflicts of Interest

The authors declare no conflicts of interest.

## Data Availability

Data sharing not applicable to this article as no datasets were generated or analysed during the current study.
